# An International Classification of Function, Disability and Health (ICF)-based investigation of movement impairment in women with pelvic organ prolapse

**DOI:** 10.4102/sajp.v75i1.472

**Published:** 2019-02-14

**Authors:** Corlia Brandt, Elizabeth C. Janse van Vuuren

**Affiliations:** 1 Department of Physiotherapy, University of the Witwatersrand, South Africa; 2 Department of Economic and Business Science, University of the Free State, South Africa

## Abstract

**Background:**

There is little evidence on movement impairment of the abdominal and pelvic floor muscles (PFM) in women with pelvic organ prolapse (POP).

**Objectives:**

The aim of this study was to determine the movement impairments and interactions between the PFM and abdominal muscles in POP.

**Method:**

The PFM and abdominal muscles of 100 conveniently sampled South African women with POP were assessed by ultrasonography, electromyography (EMG), the PERFECT scale, Sahrmann scale and a Pressure Biofeedback Unit (PBU). A demographic questionnaire determined contextual factors (exercise and medical history) and Visual Faces Scale pain intensities. Data were analysed descriptively and with Spearman and Pearson correlation coefficients.

**Results:**

Participants (59 ± 9.31 years) were mostly unemployed (80%), physically inactive (85%), with comorbidities, heart or vascular disease, hypothyroidism and depression. The mean levator hiatus at rest (56.38 mm, standard deviation [SD] 9.95), thickness (5.1 mm, SD 1.41), amount of movement (4.28 mm, SD 6.84), strength (level 1.89, SD 1.13) and endurance (4.04 s, SD 3.32) of the PFM indicated dysfunction. Median values of zero were found for the Sahrmann scale (interquartile [IQ] range [0–1]) and PBU (IQ range [0–2]) and 10.95 µV for abdominal EMG (IQ range [7.9–17.8]). Pelvic floor muscle strength, endurance, movement and EMG activity correlation was fair (*r* > 0.4, *p* < 0.001), as was PFM strength, endurance and abdominal muscle function (*r* > 0.4, *p* < 0.05).

**Conclusion:**

Movement impairment of local and global stability and mobility functions of PFM and abdominal muscles was present, as well as correlations between these functions. Addressing these impairments may affect the identified contextual factors (socio-economic, psychological and lifestyle factors) and the possible activity limitations and participation restrictions in patients with POP. Further research is needed to investigate these interactions.

**Clinical implications:**

The findings suggest that assessment and management of patients with POP might need to be based on a comprehensive neuro-musculoskeletal assessment and a holistic approach. Standardised protocols for patients with pelvic floor dysfunction (PFD) should therefore be used with caution. Randomised controlled trials should investigate patient-specific and holistic intervention approaches.

## Introduction

Pelvic floor dysfunction (PFD), such as pelvic organ prolapse (POP), is a multifactorial and under-investigated condition. The pathophysiology is complex, and the subsequent symptoms and signs affect the quality of life (QOL) of the patient and have a negative effect on global costing (Milsom 2016). Prevalence rates of 40% – 60% have been reported among women between the ages of 50 and 60 years (Wang, Hart & Mioduski 2016). Eleven per cent of women suffering from POP may need surgery, with 30% of these patients needing follow-up surgery within 2 years (Olsen et al. 1997). Many women may also have symptoms (such as urinary or faecal incontinence, pelvic pain and sexual dysfunction) for years, leading to adaptation of their lifestyle and physical activities (Wang et al. 2012). However, very little is known about the movement impairments and activity restrictions in women with POP in South Africa underlying these adaptations (Spitznagle et al. 2017).

Although the signature pedagogy of physiotherapy is defined as human movement (Jensen et al. 2017), a clinical commentary published in the Section on Women’s Health, American Physical Therapy Association, recently stated that there is not a system in place that guides the diagnosis and treatment of PFD based on movement impairments. The proposal is that diagnosis and treatment of these patients should be guided by a combination of movement-related signs, symptoms, impairments, activity and participation restrictions (International Classification of Function, Disability and Health [ICF] framework). The movement impairment should however provide the basis for diagnosis and treatment (Spitznagle et al. 2017).

For the past few years the basic mechanism underlying the prevention and treatment of POP has been described as a function of the pelvic floor muscles (PFM). The contraction of the PFM prevents descent during increases in intra-abdominal pressure, and the tone and structural support of the muscles prevent descent of the pelvic organs. The latter mechanism closes the levator hiatus and reduces the tension on the ligamentous support of the organs (Bo et al. 2007).

This role of the PFM has mainly been attributed to their strength, but further classification with regard to motor control, movement and other muscle interactions is still lacking and controversial, as is explained in the following paragraphs. When describing impairments underlying POP or PFD, the PFM should be recognised as part of the core stability and motor control mechanism. This suggests that the PFM can have a local stabiliser, global stabiliser and global mobiliser function, as they comprise both Types I and II muscle fibres. The muscles can therefore adapt to the load they are exposed to and can therefore function both as a stabiliser and mobiliser. The PFM also function in close interaction with core muscles such as the diaphragm, multifidus muscle, abdominal and other global and local stabiliser–mobiliser muscles (piriformis and obturator internis muscles), as well as the surrounding lumbar and sacro-iliac joints.

**FIGURE 1:**
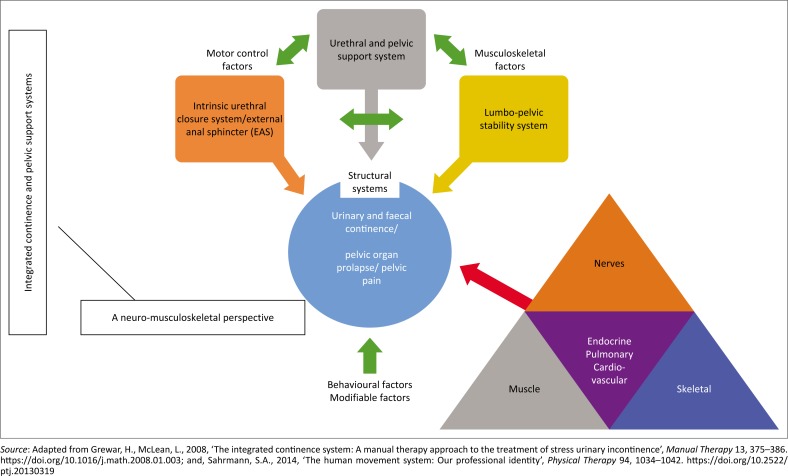
Interaction between the integrated pelvic support and continence mechanisms and the neuro-musculoskeletal system.

Motor control (movement impairments underlying the symptoms and signs) in turn can be affected by or affect social and environmental factors (QOL) according to the biopsychosocial model of rehabilitation and the ICF (Comerford & Mottram 2012). Coordinated interaction of the PFM with other muscles, as well as the articular, neural (central and peripheral factors) and connective tissue systems, may therefore play a key role in the prevention of PFD and associated impairments. This may include loss of pelvic support, lumbar and sacro-iliac joint dysfunction, loss of sphincter control and bladder and bowel symptoms, eventually affecting the QOL (Comerford & Mottram 2012) (Figure 1).

Despite the interactive role of the PFM in the motor control and core stability system, studies have isolated their investigations mainly to the strength aspect of the PFM during training and its association with pathology, without further definition of the movement impairment and how it fits into a holistic approach (Maxwell et al. 2017). Although associations have been found between activation of the PFM and the abdominal muscles, further investigation into the specific muscle roles and interactions have not been done (Sapsford et al. 2001). For example, the puborectalis (PR) muscle has an important role to minimise the levator hiatus in patients with POP and to control faecal continence. Its interactions with other neuro-musculoskeletal factors (ranging from neural to articular factors) or core muscles has however not been established. As part of the pelvic diaphragm it opposes the downward thrust of intra-abdominal pressure to generate and control intra-abdominal pressure together with the abdominal muscles. In this manner, the PFM also contribute to spinal stiffness and to force closure of the sacro-iliac joint by increased tension, counter-nutation of the sacrum as well as pelvic organ support (Pool-Goudzwaard et al. 2004).

Disturbance of any personal, environmental (such as socio-economic circumstances, lifestyle factors) or musculoskeletal factors in the pelvic or lumbar region (such as surgery, pain, weakness) may therefore lead to poorly coordinated PFM contraction and consequently urinary and/or faecal incontinence, prolapse, pain and sexual dysfunction (Comerford & Mottram 2012; Sapsford et al. 2001) (Figure 1). Differences in these domains, according to the ICF, imply that movement impairments, activity and participation restrictions might differ in different populations with POP.

The hypothesis deducted from the literature was therefore that movement impairment of the abdominal and PFM might be an interactive process between these muscle groups and part of an interactive biopsychosocial approach and core stability–motor control model. Decreased stabiliser muscle function, such as the transversus abdominus and PFM, could lead to an increased intra-abdominal pressure if not functioning effectively. It is postulated that the global abdominal mobilisers might compensate for stability and cause increased intra-abdominal pressure and therefore risk for POP. Although the model of motor control and core stability could define lumbo-pelvic neuro-musculoskeletal interactions responsible for POP, the underlying clinical evidence is still lacking and the mechanisms and interactions underlying POP not yet fully understood. Clinical data on movement impairment are needed for educational purposes and to provide clinicians with a rational basis to make decisions regarding the most appropriate and population-based management to improve the welfare of their patients (Wang et al. 2012).

The aim of this study was therefore to investigate the movement impairment of the PFM and abdominal muscles in women with POP and draw correlations between these variables.

## Methods

Patients (*n* = 120) attending appropriate clinics were approached for inclusion. One hundred women were sampled over a period of 30 months. Asian, Caucasian and mixed-race women, scheduled for pelvic floor reconstructive surgery and literate in English or Afrikaans, were included. Women had to be between 18 and 75 years of age, while pregnant women, women with Stage IV POP and women suffering from systemic neuro-musculoskeletal or psycho-sexual disorders were excluded.

All women were medically screened by a uro-gynaecologist upon their first visit to the clinic. PFD, demographic data, medical, gynaecological and exercise history were documented on a self-developed questionnaire by the uro-gynaecologist and first author. Lumbar and pelvic pain was assessed by means of the Visual Faces Scale (VFS).

### Measurement of pain

The VFS is a pain rating scale that more directly represents the feelings of participants when compared to the Visual Analogue Scale. The VFS consists of five faces with a numerical score and explanation. It is a reliable and valid method of assessment (with a median validity and test–retest reliability coefficients of 0.82 and 0.70, respectively) that has been used previously in a study investigating pain in patients with POP (Heit et al. 2002).

### Measurement of the pelvic floor muscles

A Phillips^TM^ HDIIXE was used for perineal ultrasound measurement according to the method described by Dietz (2004), Dietz, Shek and Clarke (2005) and Thompson et al. (2005). The uro-gynaecologist measured the direction, displacement and diameter of the PR muscle and levator hiatus, as well as the thickness of the perineal body upon contraction and Valsalva (Thompson et al. 2005). External anal sphincter defects were noted in the transverse plane. The intra- and inter-rater variability of perineal ultrasound has been found to be 0.77–0.91 (intra-class correlation coefficient [ICC]) (Dietz 2004).

Following the morphologic assessment, PFM strength and endurance was assessed by the PERFECT scale as described by Devreese et al. (2004) and Laycock and Jerwood (2001). This entailed establishing the maximum voluntary contraction (MVC) by means of palpation; the endurance was measured by timing the MVC up to 10 s, while the number of repetitions (up to 10) of the participant’s specific MVC was also recorded. After a 1-min rest interval, the number (up to 10) of 1-s MVCs was determined until the muscle fatigued. The scale of Devreese et al. (2004) has been demonstrated to have a high inter-observer reliability for assessment of the muscle tone (95% – 100%), and reliability coefficients between 0.75 and 1.00 have been found for the other parameters described above (Devreese et al. 2004).

A rest interval of 3 min followed the assessment by the PERFECT scale to limit muscle fatigue. A Neurotrac Myoplus^TM^ 2 (filter 19 Hz – 375 Hz) with a Periform^TM^ intravaginal probe was used for electromyography (EMG) assessment (Auchincloss & McLean 2012). The probe was inserted with the opposing electrodes in contact with the lateral vaginal walls and the reference electrode placed on the ulna, distal to the olecranon. The probe was manually supported during the test procedures to prevent it from moving or losing contact with the PFM.

The participant had to *draw in and lift the PFM* for 3 s. The test was repeated three times with a 10-s resting interval in between, and the average recorded to a precision of 0.1 µV, excluding the first second of each segment. The EMG readings were taken over a short period of time to limit normal variability of muscle behaviour or a non-specific muscle response.

Endurance was also recorded with the EMG as the time the contraction could be held above 60% of the MVC, up to a maximum of 1 min (Quartly et al. 2010).

This method of EMG measurement of the PFM, as described by Auchincloss and McLean (2012), has been found to have good to high reliability measures (ICC of 0.87 to 0.96).

With completion of the test, the reference electrode was kept in place for the EMG measurement of the abdominal muscles (Thompson et al. 2005).

### Measurement of the abdominal muscles

The method as described by Thompson et al. (2005) was used to measure the EMG activity of the internal oblique and transverse abdominus muscles. The patient was asked to slowly draw in the lower abdominal wall without any compensatory strategies. Recordings were made for 10 s with 10-s rest intervals, repeated three times and the average recorded. This method has been shown to have reasonable validity (cross-correlation of 0.96) and reproducibility (ICC of 0.9) (Lima et al. 2012).

A 1-min rest interval was allowed before measurement with the Stabiliser Pressure Biofeedback Unit (PBU) (Chattanooga^TM^, 92101D) in the same position. The PBU was placed under the lumbar lordosis and inflated to 40 mmHg. The participants were again instructed to draw in the lower abdominal wall in a similar manner. Participants were given a trial run in order to calibrate the pressure cell and determine the pretrial baseline pressure of 40 mmHg (Park & Lee 2013).

The method as described by Park and Lee (2013) and Lima et al. (2012) were followed to determine the number of correctly performed contractions at an increase of 10 mmHg, up to a maximum of 10 repetitions.

This assessment method with the PBU has been shown to have an intra- and inter-reliability of 0.74 and 0.76, respectively (Brumitt, Matheson & Meira 2013).

In the same position, the Sahrmann scale was used to test rotational control, rotational strength and sagittal strength of the abdominal muscles while monitored by the PBU (Comerford & Mottram 2012; Mills, Taunton & Mills 2005). The patient had to maintain 50 mmHg during the limb load with each level. Any pressure decrease towards 40 mmHg indicated a loss of stability into spinal extension. A pressure increase towards 60 mmHg indicated a loss of stability into spinal flexion. A change greater than 10 mmHg indicated poor control of the pelvis, and the patient was scored at the last level successfully completed. The highest level attained (with Level 5 being the highest possible score) in three trials was used for statistical analysis (Comerford & Mottram 2012; Mills et al. 2005). Stanton, Reaburn and Humphries (2004) reported a reliability coefficient of 0.95 with a standard error of mean (SEM) of 7.7% for this test.

### Data analysis

Statistical analysis was undertaken by means of the SAS software package and Excel (version 2010). Descriptive statistics, namely means, SD(s), ranges, medians and percentiles, were used to describe continuous data, and frequencies and percentages to describe categorical data. The Pearson correlation coefficient (CC) (*r*) was used for parametric continuous data correlations and the Spearman CC (*r*_s_) for non-parametric continuous and ordinal data correlations. Statistical significance was indicated by *p* < 0.05 and practical significance by means of effect size, based on the *r*-value.

### Ethical considerations

Ethical clearance was obtained from the Ethics Committee of the University of the Free State (no 25/2012). Informed consent was obtained from all the participants and permission was obtained from three uro-gynaecology clinics to conduct the study.

## Results

The sample had an average age of 59 years (standard deviation [SD] 9.13, *n* = 98) and a median body mass index (BMI) of 28.67 kg/m^2^ (interquartile [IQ] range [26.08–32.99]). Their lifestyles were characterised by an 80% unemployment rate, while 85% of the participants did not participate in any physical activity or exercise. Fifteen per cent of the women (*n* = 100) had been introduced to PFM exercises, and only 7% to core exercises; while 47% were taking medication for hypertension, 18% for cholesterol and hypothyroidism and 12% for depression. Forty-five per cent had previous gynaecological surgery, and there was a mean of 3.3 pregnancies and a median of three deliveries. Patients presented with combinations of symptoms. Eighty-six per cent of participants had a Stage III POP with overactive bladder (57%), constipation (50%), stress (37%) and urge urinary incontinence (31%), with incomplete emptying (32%) and anal incontinence (30%) being the most common symptoms. Interestingly, no participants complained of significant lumbar (mean 1.49) or pelvic pain (mean 0.99) on the VFS (maximum level of 5). Table 1 summarises the exercise and medical history.

**TABLE 1:** Results for demographic variables (categorical) (*n* = 100).

Variable	Frequency (*n*)	Percentage (*%*)
**Work**		
Manual activities or hobbies	60	60
Office work	20	20
Pensioner	20	20
**Participation in sport**	
Yes	15	15
No	85	85
**Type of exercise activities**							
Jogging	1	1
Swimming	2	2
Tennis	0	0
Walking	18	18
Weight training	1	1
Pilates and yoga	1	1
Line dance	1	1
Fishing	2	2
**Level of participation**		
Social	24	24
Provincial	0	0
National	0	0
**Comorbidities**										
Heart disease	14	14
Vascular disease	17	17
Pulmonary disease	3	3
Cancer	1	1
Allergies	21	21
Previous surgery	56	56
Inflammatory disease	19	19
Diabetes mellitus	3	3
Hypothyroidism	3	3
Depression	1	1
Psoriasis	1	1
**Medication for**																
Hypertension or angina	47	47
Hormone replacement therapy	17	17
Anti-inflammatory medication	8	8
Antidepressants	12	12
Hypothyroidism	18	18
Vitamins and minerals	9	9
Gastric ulcer	3	3
Overactive bladder	2	2
Cholesterol	18	18
Pain	7	7
Diabetes mellitus	9	9
Asthma	7	7
Constipation	1	1
Insomnia	3	3
Anticoagulant	5	5
Antihistamines	2	2
Malaria	1	1
**Smoking**	
Yes	20	20
No	80	80
**History of pelvic floor muscle exercise**	
Yes	15	15
No	85	85
**History of core or stability exercise**	
Yes	7	7
No	93	93
**Menopausal state**		
Premenopause	15	15
Perimenopause	31	31
Postmenopause	54	54
**History of pelvic or abdominal surgery**	
Yes	45	45
No	55	55

Global and local stabiliser–mobiliser functions of the PFM were affected, as indicated in Table 2. The median EMG of the PFM (22.32 µV, IQ range 11–25.6) was bordering on values found in patients without PFD (namely > 20 µV). The endurance was found to be unsatisfactory with a mean endurance of 4.04 sec (SD = 3.32) (out of a possible 10 s) when measured with the PERFECT scale. This was also reflected by an inability to repeat this contraction more than a mean of 2.8 times (SD = 2.41) (out of a possible 10 times). Endurance measurement with the EMG indicated a median of 7 s (IQ range 2–15), which is also less than endurance values of 9 s that have been found in asymptomatic women older than 40 years (Quartly et al. 2010).

**TABLE 2:** Results for pelvic floor and abdominal muscle function.

Variable	*n*	Skewness	Mean	SD	Min	Max	Median	25th percentile	75th percentile
**Sonar**									
LH at rest (mm)	98	0.475	56.379	9.953	36.0	82.3	54.30	-	-
LH during Valsalva (mm)	96	0.269	60.709	12.455	34.7	92.7	60.00	-	-
LH during contraction (mm)	97	0.852	53.186	10.239	33.0	85.5	51.40	-	-
Thickness of perineal body (mm)	95	0.535	5.102	1.413	3.0	8.4	5.00	-	-
Thickness of left PR (mm)	97	0.669	6.773	1.876	3.2	11.9	6.40	-	-
Thickness of right PR (mm)	97	0.392	6.092	1.383	2.9	9.2	5.80	-	-
Amount of movement (mm)	97	0.195	4.281	6.844	−17.1	28.0	3.90	-	-
**PERFECT scale**									
Power	100	0.136	1.890	1.136	0.0	5.0	2.0	-	-
Endurance (sec)	100	0.569	4.040	3.324	0.0	10.0	3.50	-	-
Repetitions	100	1.326	2.800	2.416	0.0	10.0	3.00	1.0	4.5
Fast contractions	100	0.152	4.690	3.446	0.0	10.0	4.00	-	-
**EMG of PFM**									
EMG at max contraction (µV)	97	1.781	22.320	17.400	2.0	88.8	17.90	11.0	25.6
Endurance with EMG (sec)	97	2.104	11.970	15.260	0.0	60.0	7.00	2.0	15.0
**Abdominal muscle function**									
Sahrmann scale	100	1.917	0.700	1.020	0.0	5.0	0.00	0.0	1.0
EMG of IO/TrA	100	1.619	13.808	8.396	2.6	50.4	10.95	7.9	17.8
PBU of TrA	100	1.584	2.100	3.560	0.0	10.0	0.00	0.0	2.0

Min, minimum; max, maximum; SD, standard deviation; LH, levator hiatus; PR, puborectalis; EMG, electromyography; IO, internal oblique muscles; TrA, transversus abdominus muscle; PBU, Pressure Biofeedback Unit; PFM, pelvic floor muscles.

The levator hiatus at rest (56.38 mm) was approximately 10 mm larger when compared to values previously found in asymptomatic subjects (aged 18–24 years), using a similar two-dimensional ultrasound technique (Dietz et al. 2005). The average movement of the PR muscle (4.28 mm) is less than half of the expected 10 mm for normal PFM function. Approximately 21% (*n* = 21) of participants also had paradoxical movement of the PR muscle and 13% (*n* = 13) of the participants had an external anal sphincter defect on the right side. The left and right PR muscle thicknesses (6.77 mm and 6.09 mm, SD = 1.87 and 1.83) were less than the expected 10 mm to 15 mm that has been found in studies investigating participants without PFD (Dietz et al. 2005). The perineal body also had a decreased thickness (5.10 mm, SD = 1.41) compared to values that have been found in normal participants (20 mm to 30 mm). An outcome measure that could also reflect poor muscle strength was the number of maximum voluntary fast contractions that was less than 50% of the optimal 10 MVCs (Table 2).

The poor physical performance was further reflected in the median values obtained for the Sahrmann scale, (Level 0, IQ range = 1), the abdominal EMG (10.95 µV, IQ range = 9.9) and the PBU readings (zero repetitions, IQ range = 2) (Table 3). Fifty-six per cent of the sample was not able to perform the local stabiliser function of the abdominal muscles correctly (measured with the PBU). Seventy-three per cent of the sample was not able to activate their abdominal muscles correctly for longer than 10 s under low load (Table 2). This also relates to the scores on the Sahrmann scale, where 57% were also not able to perform the Level 1 test correctly.

**TABLE 3:** Correlations between variables relating to movement impairments of the pelvic floor and abdominal muscles.

Variables correlated		Correlation coefficient (*r* or *r_s_*)	*df*	*p*_(two-tailed)_	Effect size based on *r*
PFM strength	PFM endurance	*r*_*s*_ = 0.677	n/a	< 0.001*	Large
PFM strength	PFM EMG activity	*r*_*s*_ = 0.63	n/a	< 0.001*	Medium
PFM endurance	PFM EMG activity	*r*_*s*_ = 0.439	n/a	< 0.001*	Medium
PFM strength	Perineal body	*r*_*s*_ = 0.03	n/a	> 0.500	Poor
PFM strength	PR thickness left PR thickness right	*r*_*s*_ = 0.0003 *r*_*s*_ = 0.078	n/a	> 0.500 > 0.200	Poor Poor
Amount of movement PR	PFM strength	*r*_*s*_ = 0.427	n/a	< 0.001*	Medium
Amount of movement PR	PFM EMG activity	*r* = 0.437	93	< 0.001*	Medium
Levator hiatus	PFM strength	*r_s_* = 0.188	n/a	> 0.050	Poor
Levator hiatus	TrA activation with PBU	*r_s_* = -0.02	n/a	> 0.500	Poor
Levator hiatus	Sahrmann level	*r_s_* = 0.079	n/a	> 0.500	Poor
Levator hiatus	PFM EMG activity	*r* = 0.036	93	> 0.050	Poor
Levator hiatus	PR thickness left PR thickness right	*r* = -0.018 *r* = 0.048	96	> 0.050	Poor
Levator hiatus	PFM endurance with EMG	*r* = -0.047	93	> 0.050	Poor
PFM strength	Sahrmann level	*r_s_* = 0.199	n/a	< 0.050*	Poor
PFM strength	TrA activation with PBU	*r_s_* = 0.125	n/a	> 0.500	Poor
PFM strength	EMG activity of TrA, IO	*r_s_* = 0.052	n/a	> 0.500	Poor
PFM EMG activity	TrA, IO EMG activity	*r* = 0.096	95	> 0.050	Poor
PFM EMG activity	Sahrmann level	*r_s_* = 0.198	n/a	> 0.050	Poor
PFM EMG activity	TrA activation with PBU	*r_s_* = 0.186	n/a	> 0.050	Poor
TrA activation with PBU	Sahrmann level	*r_s_* = 0.516	n/a	< 0.001*	Medium
PFM endurance	TrA activation with PBU	*r_s_* = 0.280	n/a	< 0.005*	Poor
Perineal body thickness	PR thickness left PR thickness right	*r* = 0.443 *r* = 0.357	94	< 0.001* < 0.001*	Medium

PFM, pelvic floor muscles; PR, puborectalis; EMG, electromyography; IO, internal oblique muscles; TrA, transversus abdominus muscle; PBU, Pressure Biofeedback Unit; n/a, not applicable.

Large effect size, 0.7–0.8; Medium effect size, 0.4–0.6.

* , statistically significant (*p* < 0.05).

Moderate to strong correlations (*r* = 0.4–0.7, *p* < 0.005) were found between local stabiliser functions of the PFM and abdominal muscles, as well as between global stabiliser and mobiliser functions of the PFM and abdominal muscles, respectively, as indicated in Table 3. Weak positive correlations were found between PFM strength and Sahrmann values (both assessing more global muscle function) (*r_s_* = 0.199, *p* < 0.05), as well as between the PFM endurance and the PBU values (both assessing more local stabiliser muscle functions) (*r_s_* = 0.28, *p* < 0.005).

## Discussion

This study is one of the first studies to investigate different aspects of PFM and abdominal muscle function in South African women with POP in order to relate it to a model of motor control, core stability and a biopsychosocial approach within an ICF framework. Considering the complexity of the pathology of PFD and POP and the interaction of contextual factors with body functions and structures (World Health Organisation 2001), a model for physiotherapeutic management of POP has been proposed where the focus should be on the movement impairments underlying and interacting with the symptoms, signs, organ dysfunction, activity limitations and participation restriction (Spitznagle et al. 2017; World Health Organisation 2001). However, the focus of this paper was only on the movement impairments of the PFM and abdominal muscles, while it identified and recognised contextual factors that most likely will interact with these movement impairments.

According to the ICF, impairments are defined as an anomaly, defect, loss or significant deviation in structures of the body. They are not synonymous with the underlying pathology but can rather be seen as a manifestation of the pathology (World Health Organisation 2001). If this definition is related to POP, it implies that the variety of bladder and bowel dysfunctions, symptoms and signs that the patients presented with in this study should be regarded as impairments. It would also be applicable to other forms of PFD, as was found by Wang et al. (2012), who found combinations of urinary, bowel and pain disorders in 2452 patients with PFD in the USA. However, underlying all of these manifestations are movement impairments of the PFM and abdominal muscles (and other neuro-musculoskeletal aspects as well), which may present in varying degrees of decreased strength, endurance, activation, movement, proprioception or coordination impairments between the muscle groups, which has not been investigated in this integrated context previously (Sapsford et al. 2001). As physiotherapists, we need to identify these movement impairments according to our signature pedagogy, in order to be able to address the activity limitations and participation restrictions they lead to.

According to the model of motor control and core stability, a motor control deficit of the stability system may lead to degenerative changes within the movement system or global imbalance and tissue overload (Comerford & Mottram 2012). It can be speculated that poor activation and local stability function of the PFM and abdominal muscles, as demonstrated by the low EMG readings, poor endurance, increased levator hiatus at rest and poor activation noted with the PBU, may lead to pathology by overloading the connective tissue system that supports the pelvic organs, eventually leading to failure of this system and POP (Bo et al. 2007). Moderate correlations between local stability functions of the PFM and abdominal muscles (measured with the PBU and Sahrmann Level 1, *r* = 0.5, *p* < 0.001, Table 3) may also indicate that addressing the local stability function of both of these muscle groups may have a preventative effect on POP.

However, the pathology of POP itself may lead to diminished proprioception and inhibition or inefficient recruitment of the slow motor units of both the global and local stability muscles (Comerford & Mottram 2012). This is because of the traction that is placed on the musculature and supporting ligaments. Dysfunction of the local stability component was indicated previously, while dysfunction of the global stability component was indicated by the slow, decreased eccentric movement observed in the PR muscle under low load, as well as the majority of the sample that was not able to perform better than a Level 3 on the Sahrmann scale. Sahrmann Level 1 measures the low load rotational control of the abdominal muscles, whereas rotational strengthening under higher loads (global stability function where the external oblique muscles are activated) is measured by Levels 2 and 3 (Comerford & Mottram 2012). This might explain the moderate positive correlation that was found between low load activation of the abdominal muscles with the PBU (which is more an indication of local stability function) and measurement on the Sahrmann scale (which is usually more an indication of global stability function when testing a Level 2 or higher) (*r* = 0.5, *p* < 0.001, Table 3).

Predicted by most motor control models, the global mobilisers may show excessive dominance or dysfunctional patterns in reaction to poor stabilising function or pathology, leading to further inhibition of the slow motor unit recruitment (Comerford & Mottram 2012). This can often be observed in the form of abdominal bracing in patients with POP, which increases the intra-abdominal pressure and worsens the prolapse (Sapsford 2004). However, strength assessment of the abdominal muscles with the Sahrmann scale showed that very few participants could reach a Level 4 or 5 strength, while the strength assessment of the PFM with the PERFECT scale also indicated values below normal. The thickness of the muscles (which in some instances can be related to muscle strength; Jull et al. 2015) also demonstrated atrophy, when compared to normal values. However, no correlations were found between the thickness of the PFM and the PFM strength in our study (*r* = 0.0 and 0.08, respectively; *p* < 0.05) (Table 3), which could question the appropriateness of muscle thickness as an indicator of strength.

As the perineal body is considered an important structural support for the pelvic organs, together with the PFM strength, it might be important to seek methods to increase the thickness of the PFM, as the correlation with the perineal body thickness was fair (*p* < 0.001, *r* = 0.4, Table 3). It must also be considered that other global musculature forming part of the core (such as the gluteus maximus muscle, internal and external oblique muscles) was not assessed and might have shown compensation strategies in reaction to the poor stability function observed.

No negative correlations were found between mobiliser and stability functions of the PFM and abdominal muscles in our study. This finding therefore suggests that training of the mobiliser function may not necessarily adversely affect the stabiliser muscle function (or vice versa) in these two muscle groups. The moderate positive correlations between the PFM strength, endurance and activity (*r* = 0.6 and 0.3, *p* < 0.001) (Table 3) also indicated that addressing all three of these aspects may contribute to improved pelvic organ support by means of increased tone, narrowing of the levator hiatus and increased structural support. Therefore, according to our study’s findings, the problem seems not to be an imbalance between the local and global stability function of the muscles investigated (as both aspects were poor). Correct recruitment and activation patterns might rather need to be considered.

The findings of poor muscle function could also be related to personal factors (Table 1) such as older age and menopausal status, which can lead to atrophy (Zhu et al. 2005). The fact that only 15% of participants had ever done PFM exercises and even fewer (7%) had been exposed to any type of core exercises could also be a reason for the poor muscle function. Low levels of physical activity have previously been linked to increased rates of incontinence (Townsend et al. 2008). Furthermore, the contextual factors of the participants, such as the high unemployment rate and presence of comorbidities such as depression and cardiovascular disease, also indicate aspects within the motor control model that could affect the neuro-musculoskeletal interaction at a higher central nervous system level.

Khan et al. (2013) indicated a strong correlation between the severity of anxiety and depression and the severity of PFD. In addition to the findings of our study, an investigation into the extent of the interaction between contextual and neuro-musculoskeletal factors in patients with PFD and POP would therefore be a recommendation for future research.

**FIGURE 2:**
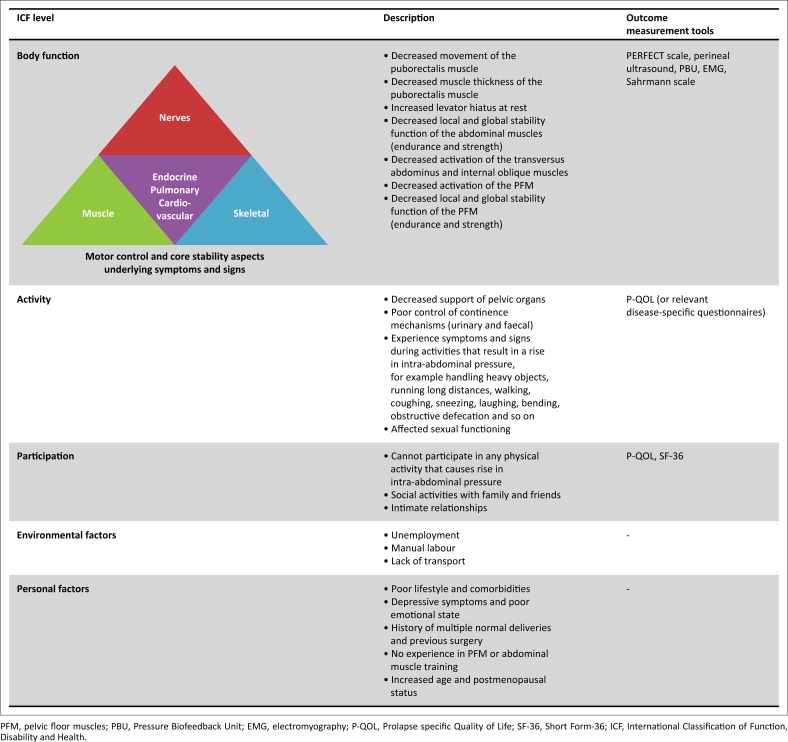
Proposed summary of the findings regarding movement impairment of the abdominal and pelvic floor muscles in women with pelvic organ prolapse within an ICF and motor control framework.

Defining movement impairment according to the interactive processes of motor control and core stability within an ICF framework (contextual and personal factors) may therefore imply that the components underlying the pathology, limitations and restrictions will be different in different populations and individuals (World Health Organisation 2001). This concept is demonstrated in Figure 2.

Although all measuring instruments used in this study, such as surface EMG, have been shown to be valid and reliable, techniques such as needle EMG, three- or four-dimensional ultrasound or MRI might yield more advanced and accurate measurements. Assessment of the timing and coordination of muscle contraction as proposed by Devreese et al. (2004) may further improve the methodology and understanding of the complexity of the interactions and mechanisms underlying the movement impairments of PFD and POP. Future research should also consider comparing results with a control group; however, performing internal examinations on healthy volunteers has posed ethical challenges in previous pilot studies.

## Conclusion

This study made a unique contribution to the understanding of POP by investigating and correlating different aspects of movement impairment of the abdominal and PFM function within an ICF framework and motor control and core stability model. Although this interaction has been suggested before, it has not been substantiated clinically nor integrated into an ICF framework. Movement impairment of both local and global stability and mobility functions of the PFM and abdominal muscles, as well as correlations between these functions underlying the symptoms and signs, were present in this sample of women with POP. Addressing these impairments may therefore affect the activity limitations, participation restrictions and some contextual factors in individuals with POP. These findings suggest that assessment and management of patients with POP might need to be based on a comprehensive neuro-musculoskeletal assessment and a holistic approach. Standardised protocols for patients with PFD should therefore be used with caution, and further investigation is needed to substantiate the suggestions emanating from this study.
